# Characterization of a novel bacteriophage endolysin (LysAB1245) with extended lytic activity against distinct capsular types associated with *Acinetobacter baumannii* resistance

**DOI:** 10.1371/journal.pone.0296453

**Published:** 2024-01-02

**Authors:** Rosesathorn Soontarach, Potjanee Srimanote, Buppa Arechanajan, Alisa Nakkaew, Supayang Piyawan Voravuthikunchai, Sarunyou Chusri

**Affiliations:** 1 Faculty of Science, Center of Antimicrobial Biomaterial Innovation-Southeast Asia, Prince of Songkla University, Songkhla, Thailand; 2 Faculty of Medicine, Department of Internal Medicine, Division of Infectious Diseases, Prince of Songkla University, Songkhla, Thailand; 3 Faculty of Allied Health Sciences, Graduate in Biomedical Sciences, Thammasat University, Pathum Thani, Thailand; 4 Faculty of Science, Division of Biological Science, Program in Molecular Biology and Bioinformatics, Prince of Songkla University, Songkhla, Thailand; Nitte University, INDIA

## Abstract

Capsular polysaccharides are considered as major virulence factors associated with the ability of multidrug-resistant (MDR) *Acinetobacter baumannii* to cause severe infections. In this study, LysAB1245, a novel bacteriophage-encoded endolysin consisting of a lysozyme-like domain from phage T1245 was successfully expressed, purified, and evaluated for its antibacterial activity against distinct capsular types associated with *A*. *baumannii* resistance. The results revealed a broad spectrum activity of LysAB1245 against all clinical MDR *A*. *baumannii* isolates belonging to capsular type (KL) 2, 3, 6, 10, 47, 49, and 52 and *A*. *baumannii* ATCC 19606. At 2 h following the treatment with 1.7 unit/reaction of LysAB1245, more than 3 log reduction in the numbers of bacterial survival was observed. In addition, LysAB1245 displayed rapid bactericidal activity within 30 min (nearly 3 log CFU/mL of bacterial reduction). Thermostability assay indicated that LysAB1245 was stable over a broad range of temperature from 4 to 70°C, while pH sensitivity assay demonstrated a wide range of pH from 4.5 to 10.5. Furthermore, both minimal inhibitory concentration (MIC) and minimal bactericidal concentration (MBC) of LysAB1245 against all MDR *A*. *baumannii* isolates and *A*. *baumannii* ATCC 19606 were 4.21 μg/mL (0.1 unit/reaction). Conclusively, these results suggest that LysAB1245 possesses potential application for the treatment of nosocomial MDR *A*. *baumannii* infections.

## Introduction

Recently, an outbreak of *A*. *baumannii* infections in coronavirus disease 2019 (COVID-19) patients has been reported [1–3], resulting in increased morbidity and mortality rates as well as high treatment costs. This emerging pathogen is responsible for several healthcare-associated infections, such as bacteremia, ventilator-associated pneumonia, urinary tract infections, burn and wound infections, and meningitis [4–7]. Nosocomial *A*. *baumannii* infections usually correlate with the production of capsular polysaccharide (CPS), which plays an important role in bacterial pathogenesis by protecting from environmental stresses, antimicrobial penetration, and host immune responses [8,9]. In 2019, more than 100 distinct capsular types (KL) of *A*. *baumannii* were discovered, with the variations in K unit structures and sugar composition [10]. In a previous study, three capsular genotypes, including KL6, 10, and 47, showed a frequency more than 10% among *A*. *baumannii* isolates from three tertiary care hospitals in Thailand [11]. Moreover, carbapenem-resistant *A*. *baumannii* isolates belonging to KL2, 10, 22, and 52 showed higher incidence and mortality rates than isolates belonging to other KL groups [12]. Nowadays, isolates of *A*. *baumannii* rapidly develop resistance to several currently employed antibiotics, including carbapenems, aminoglycosides and polymyxin [13–18]. Therefore, novel and effective antibacterial agents targeting emerging antibiotic-resistant *A*. *baumannii* strains are urgently required.

Bacteriophages (phages) are known as natural enemies of bacteria that have no harmful effects on the human microbiome. Therefore, the use of bacteriophages has been considered as an alternative therapeutic option for drug-resistant *A*. *baumannii* infections [19,20]. Moreover, phage-encoded enzymes, such as endolysins, are are effective in eradicating or reducing antibiotic-resistant pathogenic bacteria [21,22]. Endolysins are peptidoglycan lytic enzymes capable of breaking down bacterial cell walls, and these enzymes can be used as recombinant proteins to attack invading bacterial cells. Endolysins are widely used as antibacterial agents due to their major advantages over phages and antibiotics, including rapid killing activity, high efficiency, and a broad spectrum of lytic activity against pathogenic bacteria without showing toxicity on human cells [23–25]. In addition, the C-terminal cell wall binding domain of phage-encoded endolysin is responsible for rapid kinetic, which is highly specific to the peptidoglycan of bacterial cells and poses a low risk of resistance development [26,27].

In our previous study, a novel virulent phage T1245 specifically infecting *A*. *baumannii* with KL10 was isolated, characterized, and subjected to biological property tests and whole genome sequencing [28]. However, it is necessary to evaluate the antibacterial activity of its endolysin. In this study, the gene-encoding endolysin of phage T1245, named LysAB1245 was cloned into the expression vector, expressed, and produced as purified proteins. Purified endolysin, LysAB1245 was tested for its catalytic properties against different major capsular types associated with *A*. *baumannii* resistance. Overall, this study aimed to produce a protein suitable for further development as an alternative antibacterial agent against MDR *A*. *baumannii* isolates belonging to common capsular types.

## Materials and methods

### Bacteria, bacteriophage, and culture conditions

The bacterial strains, phage, plasmids, and primers used in this study are listed in Table 1. All MDR *A*. *baumannii* strains belonged to sequence type 2 with different major capsular types, including KL2, 3, 6, 10, 47, 49, and 52. All bacteria were inoculated in Luria Bertani (LB) broth or LB agar (Difco Laboratories, Detroit, MI, USA) at 37°C and maintained in 20% glycerol (v/v) at -80°C for long-term storage. This study was approved by the Human Research Ethics Committee (HREC) of the Faculty of Medicine at Prince of Songkla University (reference number: 64–284–14–1).

**Table 1:** Bacterial strains, bacteriophage, plasmids, and oligonucleotide primers.

Strain, plasmid, phage, or primer	Relevant characteristic(s), description, or sequence	Source or reference
**Strains**		
*A*. *baumannii* ABMYH-1245	Multidrug-resistant, clinical isolate with KL10; primary host bacteria of phage T1245	[11]
*A*. *baumannii* ABAPSP-55	Multidrug-resistant, clinical isolate with KL10	[11]
*A*. *baumannii* ABAPSP-64 *A*. *baumannii* ABMYSP-109	Multidrug-resistant, clinical isolate with KL10 Multidrug-resistant, clinical isolate with KL10	[11]
*A*. *baumannii* ABMYSP-101	Multidrug-resistant, clinical isolate with KL10	[11]
*A*. *baumannii* ABMYSP-182	Multidrug-resistant, clinical isolate with KL10	[11]
*A*. *baumannii* AB1039	Multidrug-resistant, clinical isolate with KL2	[11]
*A*. *baumannii* AB3396	Multidrug-resistant, clinical isolate with KL2	[11]
*A*. *baumannii* ABJNH-403	Multidrug-resistant, clinical isolate with KL3	[11]
*A*. *baumannii* ABMYSP-185	Multidrug-resistant, clinical isolate with KL6	[11]
*A*. *baumannii* ABMYSP-210	Multidrug-resistant, clinical isolate with KL6	[11]
*A*. *baumannii* ABMYSP-216	Multidrug-resistant, clinical isolate with KL6	[11]
*A*. *baumannii* ABMYSP-419	Multidrug-resistant, clinical isolate with KL6	[11]
*A*. *baumannii* ABMYH-1033	Multidrug-resistant, clinical isolate with KL6	[11]
*A*. *baumannii* AB15	Multidrug-resistant, clinical isolate with KL47	[11]
*A*. *baumannii* ABAPP-61	Multidrug-resistant, clinical isolate with KL47	[11]
*A*. *baumannii* AB724	Multidrug-resistant, clinical isolate with KL49	[11]
*A*. *baumannii* AB2792	Multidrug-resistant, clinical isolate with KL49	[11]
*A*. *baumannii* ABMYSP-21	Multidrug-resistant, clinical isolate with KL52	[11]
*A*. *baumannii* ABMYSP-444	Multidrug-resistant, clinical isolate with KL52	[11]
*E*. *coli* Top10	Laboratory strain for TA cloning use	Invitrogen, San Diego, USA
*E*. *coli* BL21 (DE3	Laboratory strain for protein expression	Invitrogen, San Diego, USA
**Plasmids**		
pGEM-T-easy	3,015-bp *E*. *coli* vector, Ampr, Plac, lacZ	Promega, San Diego, USA
pET30b(+)	Expression vector; 5,421-bp *E*. *coli* vector, Kmr, PT7, His-Tag	Novagen, Wisconsin, USA
**Phages**		
*A*. *baumannii* phage T1245	Accession No. ERS3583556	[28]
**Primers**		
Forward primer: FP-EcoRIEndo	GAATTCGATGATTCTGACTAAAGACGG	This study
Reverse primer: RP-XholEndo	CTCGAGTAAGCTCCGTAGAG	This study

### Bioinformatics analysis

The whole genome of phage T1245 was deposited in GenBank under accession number ERS3583556. The gene-encoding endolysin (LysAB1245) in phage T1245 was blasted in the NCBI protein database (https://blast.ncbi.nlm.nih.gov/Blast.cgi). Amino acid sequences of LysAB1245 and other reported *Acinetobacter* phage endolysins were aligned using ClustalOmega multiple sequence alignment (https://www.ebi.ac.uk/Tools/msa/clustalo/).

### Construction of LysAB1245 expression vector

LysAB1245*-*encodin*g* gene in phage T1245 was amplified by polymerase chain reaction (PCR). Gene information and the PCR primers are listed in Table 1. LysAB1245, 569-bp PCR products were purified using the GenepHlowTM Gel/PCR Cleanup Kit (Geneaid, Taiwan). Purified LysAB1245 gene was cloned into the pGEM-T cloning vector according to standard procedures [29]. Ligation reaction was performed and transformed into *Escherichia coli* Top 10 by heat shock method. The transformed cells were plated onto LB agar plates containing ampicillin (100 μg/mL), isopropyl b-D-1 thiogalactopyranoside (IPTG), and X-Gal. Plates were incubated at 37°C for 18 h. White-positive colonies were selected and verified using PCR and sequencing. Purified LysAB1245 genes were digested with EcoRI and Xhol, purified from the agarose gel using a QIAquick gel extraction kit (Qiagen, Hilden, Germany), and assembled into EcoRI/Xhol digested pET30b(+). The resulting plasmids (pET30b(+)-LysAB1245) were transformed into an *E*. *coli* BL21 (DE3) strain for over-expression. The transformed cells were plated onto agar plates containing kanamycin (50 μg/mL) and incubated overnight at 37°C for 16–18 h. Glycerol stocks were prepared from positive clones and sequencing was performed to confirm LysAB1245 expression.

### Expression and purification of endolysin LysAB1245

An ExiProgen automated protein synthesis system (ExiProgen^TM^, Bioneer, Korea) with cell-free protein synthesis and magnetic bead-based His-Tag affinity purification was used to express and purify endolysin LysAB1245. Briefly, 6 μg of plasmid DNA (pET30b(+)-LysAB1245*)* was prepared for LysAB1245 synthesis. Ten microliters of DNA was added to the reaction well of the ExiProgen^TM^ EC1 protein synthesis kit’s protein expression cartridge. The reaction was performed using *E*. *coli* cellular lysate and Bioneer’s master mix for the transcription and translation of coding sequence to protein and purification of the target protein. After 6 h, 250 μL of purified protein samples were collected from each elution tube. Bradford assay using bovine serum albumin was performed to determine the concentration of LysAB1245. Endolysin protein synthesis has an efficiency of over 30% yield, according to the manufacturer’s maximum efficacy.

### SDS-PAGE and western blot analysis

Sodium dodecyl sulfate-polyacrylamide gel electrophoresis (SDS-PAGE) and western blot analyses were performed to assess the purity of endolysin LysAB1245. Samples collected from ExiProgen including purified protein in elution tubes, unbound, expression, and washing were mixed with sample buffer (62.5 mM Tris–HCl, pH 6.8, containing 5% 2-mercaptoethanol, 2% sodium dodecyl sulfate, 10% glycerol, and 0.01% bromophenol blue) and heated for 5 min in boiling water. All samples were separated using 12% SDS-PAGE and blotted onto a 0.45-μm nitrocellulose membrane (Bio-Rad).

For the immunodetection of a 6×His-tagged protein, the membrane was blocked with 3% bovine serum albumin in phosphate-buffered saline (10 mM PBS; pH 7.4) for 18 h, followed by incubation with a mouse anti-His-tag antibody (1:3000) (Bio-Rad, Hercules, CA, USA) for 1 h. After three washes with PBS containing Tween 20 (PBST), the membranes were incubated with alkaline phosphatase-labeled goat anti-mouse IgG antibody (1:3000) (KPL, Gaithersburg, MD, USA) for 1 h. After four washes with PBST, the blot was developed using 5-bromo-4-chloro-3-indolyl-phosphate (BCIP) and nitro blue tetrazolium (NBT) (Sigma, Deisen- hofen, Germany).

### Effects of LysAB1245 on MDR *A*. *baumannii* ABMYH1245

The effects of purified LysAB1245 were examined on a primary host of phage T1245 according to the protocol of Lai et al. (2011) [30], with slight modifications. Briefly, the log phase of *A*. *baumannii* AMMYSP-1245 was grown in tryptic soy broth (TSB; Difco) and adjusted to 10^5^ colony-forming units (CFU/mL). The bacterial cells were centrifuged at 9,000 rpm for 5 min and the supernatant was discarded. Thereafter, bacterial pellets were treated with 50 μL of LysAB1245 (134.71 μg/mL) or 10 mM of PBS (as a control) followed by incubation at 37°C under constant shaking at 150 rpm. Samples were collected at 0, 2, and 24 h, and the log CFU/mL was calculated. Data obtained from two independent experiments performed in triplicate are presented as mean ± standard deviation (SD). Significant differences between groups were determined using one-way analysis of variance (ANOVA), followed by Tukey’s multiple comparison test. Statistical significance was set at 99% confidence interval (*p* < 0.05). One unit of enzyme activity was defined as the amount of enzyme required to kill bacterial cells at 2 logs per 2 h.

### Determination of the lytic range of LysAB1245

The antibacterial activity of LysAB1245 was determined against 19 isolates of MDR *A*. *baumannii* belonging to seven different major capsular types (KL2; 2 isolates, KL3; 1, KL6; 5, KL10; 5, KL47; 2, KL49; 2, and KL52; 2) and ATCC 19606. Briefly, the bacterial pellets at 10^5^ CFU/mL were resuspended with 50 μL of LysAB1245 (1.7 unit/reaction) or PBS and incubated at 37°C with shaking at 150 rpm. Samples were collected at 0, 2, and 24 h and calculated the number of log CFU/mL. Data obtained from two independent experiments performed in triplicate are presented as mean ± SD.

### Sensitivity of LysAB1245 to temperature and pH

Thermal and pH stability test for endolysin LysAB1245 were examined. Briefly, LysAB1245 (0.4 unit/reaction) was incubated at six different temperatures (4, 25, 37, 50, 60, and 70°C) for 30 min. Subsequently, the cell pellets of *A*. *baumannii* AMMYSP-1245 (10^5^ CFU/mL) were resuspended with LysAB1245 from various temperatures and incubated at 37°C with shaking (150 rpm) for 24 h.

For pH sensitivity assay, the pH of LysAB1245 was adjusted using PBS with different pH values (4.5, 5.5, 7.4, 8.5, and 10.5), followed by incubation at 37°C. After 30 min, the pH of LysAB1245 was adjusted to 7.4 and incubated at 37°C with shaking for 24 h. For both experiments, a mixture of bacteria and PBS (pH 7.4) at 37°C was served as the control group. The number of log CFU/mL was determined and calculated the percentage of bacterial reduction. Each experiment was performed in triplicate with two independent replicates.

### Kinetic analysis of LysAB1245

A mixture of MDR *A*. *baumannii* ABMYH-1245 (at 10^5^ CFU/mL) and LysAB1245 (at 1.7 unit/reaction) or PBS (as a control) was incubated at 37°C with shaking at 150 rpm. The samples were withdrawn at 0, 15 min, and 30-min intervals for 0.5–2.5 h and the number of log CFU/mL was determined. Data obtained from two independent experiments performed in triplicate are presented as mean ± SD.

### Antimicrobial activity of LysAB1245

The minimum inhibitory concentration (MIC) of LysAB1245 was determined using the broth microdilution method, according to the Clinical and Laboratory Standard Institute (CLSI) guidelines [31]. Briefly, a single colony of 20 clinical MDR *A*. *baumannii* isolates belonging to KL 2, 3, 6, 10, 47, 49, and 52 and *A*. *baumannii* ATCC 19606 was grown in Mueller-Hinton broth (MHB; Difco) and incubated at 37°C with shaking until the cells reached the logarithmic phase. Endolysin LysAB1245 (134.71 μg/mL) was serially diluted (1:2) in microtiter plate (50 μL/well). Subsequently, 50 μL of *A*. *baumannii* culture with approximately 10^6^ colony-forming units (CFU/mL) was inoculated to the microplate, containing different diluted LysAB1245 and further incubated at 37°C for 18 h. MIC was defined as the of the antibacterial agent that inhibited the visible growth of bacteria, while minimal bactericidal concentration (MBC) was defined as the lowest concentration of the antibacterial agent required to kill bacteria. The experiment was performed in triplicate in two independent experiments.

## Results and discussion

### Characterization of endolysin LysAB1245

The endolysin gene of phage T1245, named LysAB1245 contains 558 base pairs and 185 amino acids. BLAST analysis showed that the LysAB1245 gene had 98.92% sequence similarity to putative chitinase-like endolysin from *Acinetobacter* phage phiAB6 (accession no. YP_009288673.1). The results of conserved domain analysis using the Pfam database revealed that amino acids of LysAB1245 contain lysozyme-like (*N*-acetyl-β-D-muramidase) domains between residues 79 and 136, which are the catalytic domains of LysAB1245. The catalytic activities of purified endolysin LysAB1245 are attributable to glycosidases that cleave β-1,4-*N*-acetyl-D-glucosamine bonds between *N*-acetylmuramic acid and *N*-acetylglucosamine in glycan chains [32]. Previously, phage endolysins from different host genera and species such as PlyE146 from *E*. *coli* phage [33], LysSS from *Salmonella enterica* phage [20] and LysAB2, PlyAB1, LysABP-01, and Ply6A3 from *A*. *baumannii* phages [24,30,34,35] were studied their catalytic activities, which belong to glycosidase hydrolase family.

**Fig 1:**
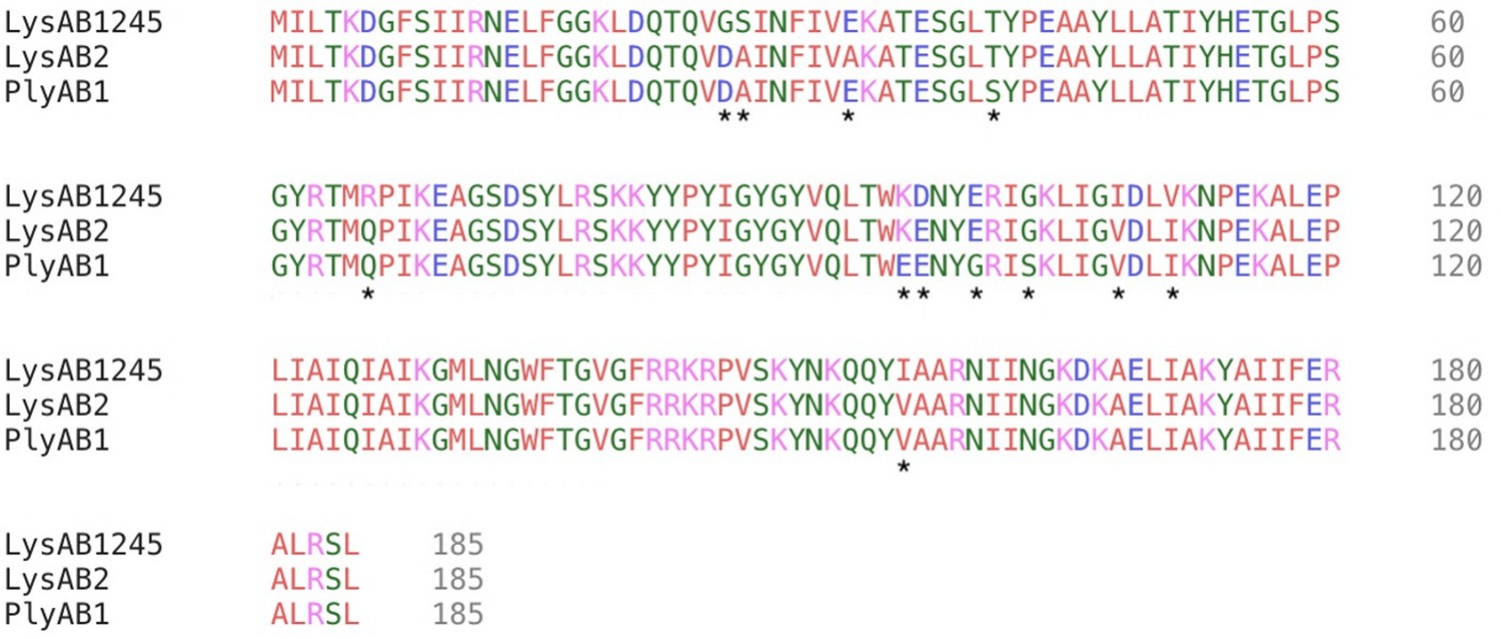
Amino acid sequence alignment using Clustal Omega multiple sequence alignment. The multiple sequence alignments of three phage endolysins revealed similar and dissimilar amino acids of LysAB1245, LysAB2 (accession no. ADX62345), and PlyAB1 (accession no. YP_008058242). Amino acid polymorphisms are indicated by asterisks (*).

Multiple sequence alignments of LysAB1245 with two other reported *A*. *baumannii* phage endolysins showed high similarity in the domain region with 12 amino acid polymorphisms identified (Fig 1). However, *in silico* sequence analysis indicated that six mutations (amino acids 96, 97, 100, 103, 108, and 111) were founded in the conserved domain, which play a vital role in enhancing the catalytic function of the enzyme

### Cloning, expression, and purification of endolysin LysAB1245

**Fig 2:**
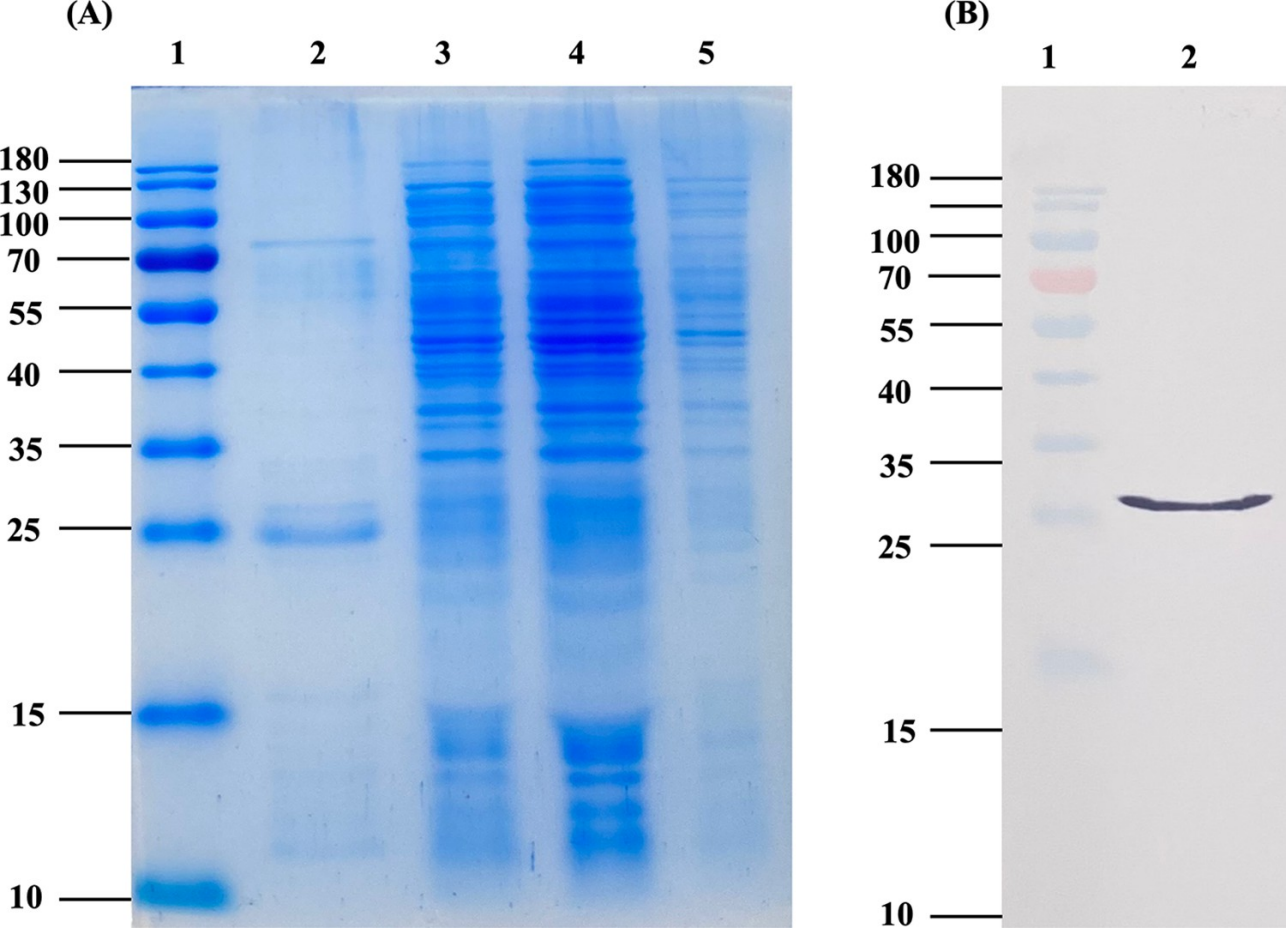
**SDS-PAGE analysis of LysAB1245 (A)**. Lane 1, molecular size marker; lane 2, purified LysAB1245; lane 3, expression sample; lanes 4, washed sample; lanes 5, unbound sample. **Western blot analysis of LysAB1245 (B)**. Lane 1, molecular size marker; lane 2, purified LysAB1245.

SDS-PAGE results indicated that LysAB1245 was effectively expressed and purified using the ExiProgen protein synthesis system (Fig 2A). Western blot analysis using specific His-tagged antibodies revealed an expected size of approximately 26 kDa (Fig 2B). Moreover, the concentration of purified LysAB1245 was approximately 134.71 μg/mL (S1 Fig).

### Lytic effects of purified endolysin LysAB1245 on *A*. *baumannii* ABMYH-1245 cells

**Fig 3:**
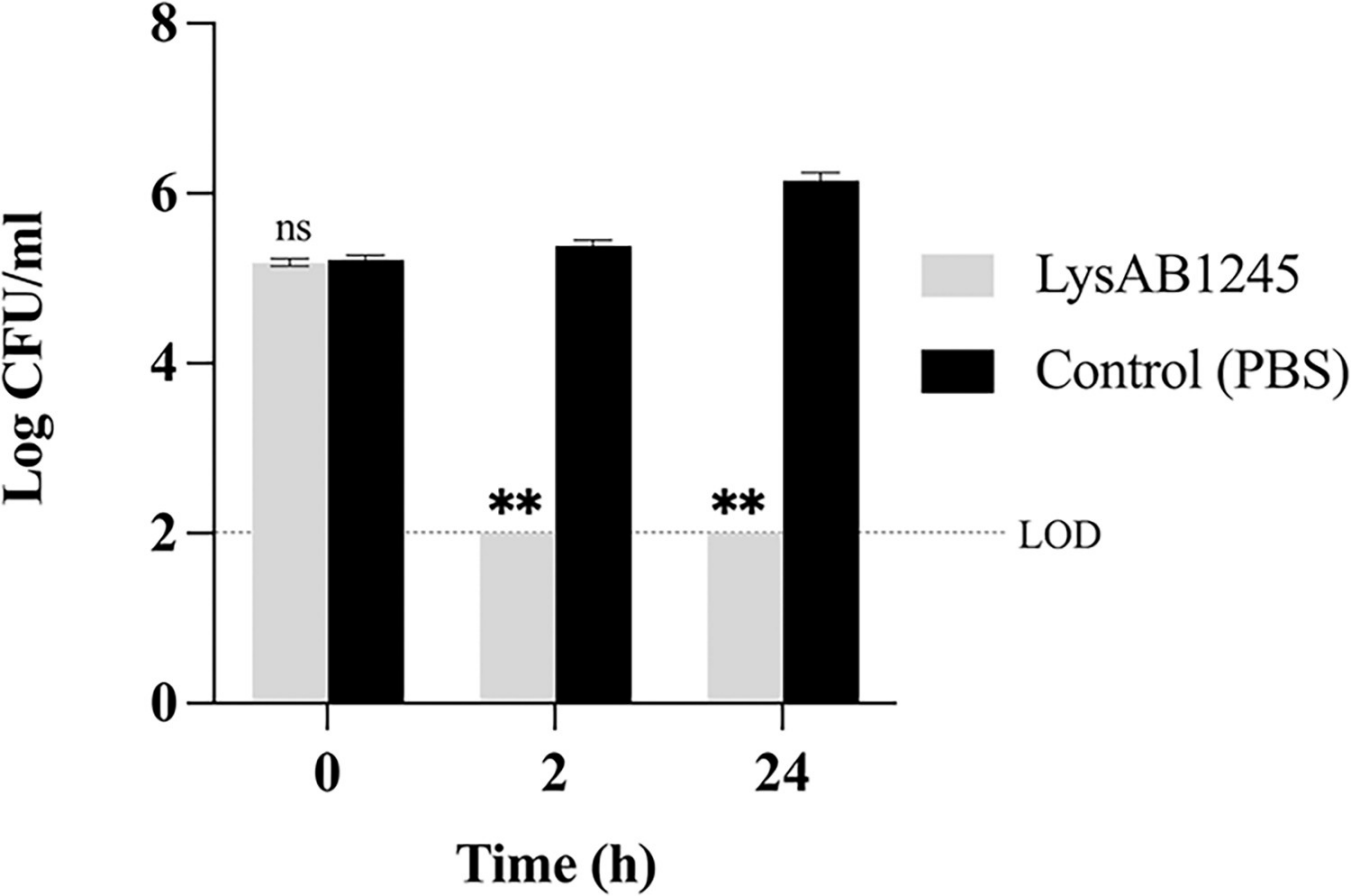
The antibacterial activity of purified endolysin LysAB1245 on *A*. ***baumannii* ABMYSP-1245 cells.** Bacterial pellets (10^5^ CFU/mL) were treated with LysAB1245 (134.71 μg/mL). A significant reduction in bacterial growth was compared with control, **P < 0.01, and ns means non-significant. Two independent experiments were performed in triplicate and error bars represent the standard deviation. Limit of detection (LOD) for surviving bacterial cells was 2 log CFU/mL.

Treatment with LysAB1245 at 134.71 μg/mL significant decreased (*P* <0.01) the growth of *A*. *baumannii* ABMYSP-1245 when compared with control at 2 h and 24 h (Fig 3). At 2 h and 24 h, LysAB1245 reduced the viability of *A*. *baumannii* cells up to 3.39 log CFU/mL (>99.9% reduction) and 4.16 log CFU/mL (>99.99% reduction), respectively, compared with control (S2 Fig). Compared with other phage endolysins such as LysSP1 (*Salmonella* phage) and LysPN09 (*Pseudomonas* phage), LysAB1245 exhibited efficient bactericidal activity, even in the absence of outer membrane permeabilisers [36,37]. Generally, endolysins exert their effects against Gram-positive bacteria by binding directly to the cell walls. In contrast, the presence of an outer membrane can prevent the entry of several antimicrobials into Gram-negative bacterial cells. The mechanism of LysAB1245 on Gram-negative bacteria could be attributed to the highly positively charged region at its C-terminus, which has the potential to destabilize the outer membrane of Gram-negative bacteria. Consequently, the N-terminal enzymatic domain can penetrate the peptidoglycan layer and induce bacterial lysis [38]. In addition, Düring et al. reported that helix-forming amphipathic peptides at the C-terminus of T4 lysozyme can interact with negatively charged lipopolysaccharides in Gram-negative bacteria, resulting in antimicrobial activities [39].

### Lytic spectrum of purified endolysin LysAB1245 on MDR *A*. *baumannii* isolates with major different capsular types

**Fig 4:**
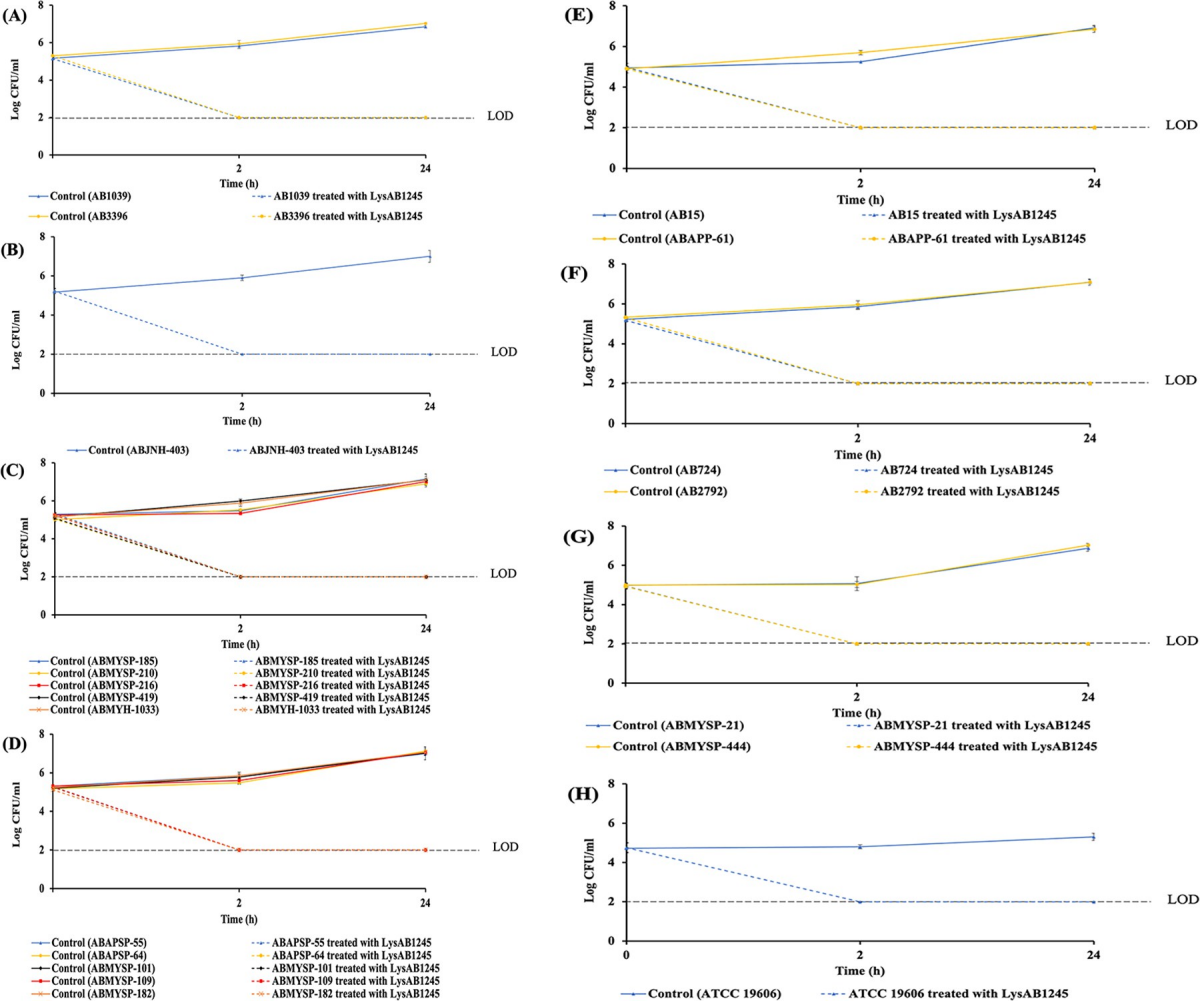
The lytic spectrum of LysAB1245 on multi-drug resistant (MDR) *A*. ***baumannii* isolates**. Bacterial pellets (10^5^ CFU/mL) were treated with LysAB1245 (1.7 unit/reaction). *Acinetobacter baumannii* with KL2 **(A)**, KL3 **(B)**, KL6 **(C)**, KL10 **(D)**, KL47 **(E)**, KL49 **(F)**, and KL52 **(G)** and ATCC 19606 **(H)**. Two independent experiments were performed in triplicate and error bars represent the standard deviation. Limit of detection (LOD) for surviving bacterial cells was 2 log CFU/mL.

Recently, a newly isolated lytic phage targeting the MDR *A*. *baumannii* isolates, phage T1245 was isolated and characterized. However, phage T1245 specifically infects only *A*. *baumannii* with KL10 and KL3 isolates. Capsular structure has been recognized as an important virulence factor among *A*. *baumannii* strains [40]. LysAB1245 was further investigated whether it could lyse MDR *A*. *baumannii* isolates belonging to major capsular types identified in the Thai collection. The results of lytic spectrum revealed that LysAB1245 not only killed *A*. *baumannii* with KL10 but also lysed all the tested clinical MDR *A*. *baumannii* isolates with KL2, 3, 6, 47, 49, and 52 and *A*. *baumannii* ATCC 19606. A more than 3-log reduction (>99.9% reduction) in the viability of all tested MDR *A*. *baumannii* isolates belonging to KL2, 3, 6, 10, 47, 49 and 52 and *A*. *baumannii* ATCC 19606 was observed when treated with LysAB1245 at 1.7 unit/reaction, compared with control at 2 h (Fig 4). Moreover, no re-growth of any *A*. *baumannii* isolates was observed after 24 h of treatment with LysAB1245. Notably, MDR *A*. *baumannii* with KL2 and KL49 which were not lysed by any isolated phages from previous study, were killed by endolysin LysAB1245.

### Stability of LysAB1245 under various temperature and pH conditions

**Fig 5:**
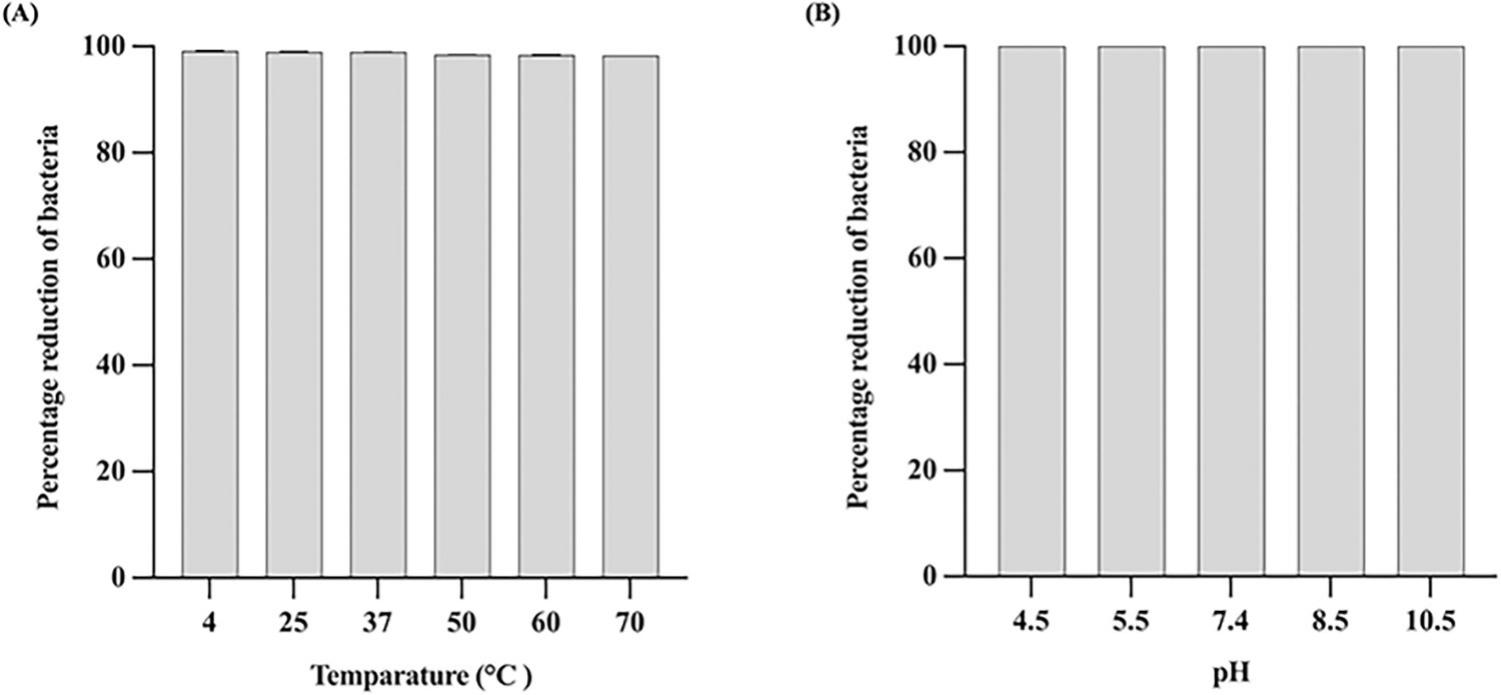
The stability of purified endolysin LysAB1245 at various temperatures and pH conditions. Bacterial pellets (10^5^ CFU/mL) were mixed with LysAB1245 at 4, 25, 37, 50, 60, and 70°C (A) and pH 4.5, 5.5, 7.4, 8.5, and 10.5 (B). The percentage of bacterial reduction was compared with the control. The experiment was performed in triplicate and error bars represent the standard deviation.

Thermal and pH stability are desirable properties of antibacterial agents during storage. Therefore, the stability of LysAB1245 under different temperatures and pH conditions was examined. LysAB1245 remained stable and highly bactericidal at temperatures ranging from 4 to 70°C (>99% reduction in bacterial cells when compared with the control) (Fig 5A). Additionally, the activity of LysAB1245 was relatively stable to pH changes over a range from 4.5 to 10.5 (Fig 5B) (S2 Fig). The results indicated that LysAB1245 could be used as a potential alternative antibacterial agent due to its stable activity across a broad range of thermal and pH conditions.

### Kinetic analysis of LysAB1245

**Fig 6:**
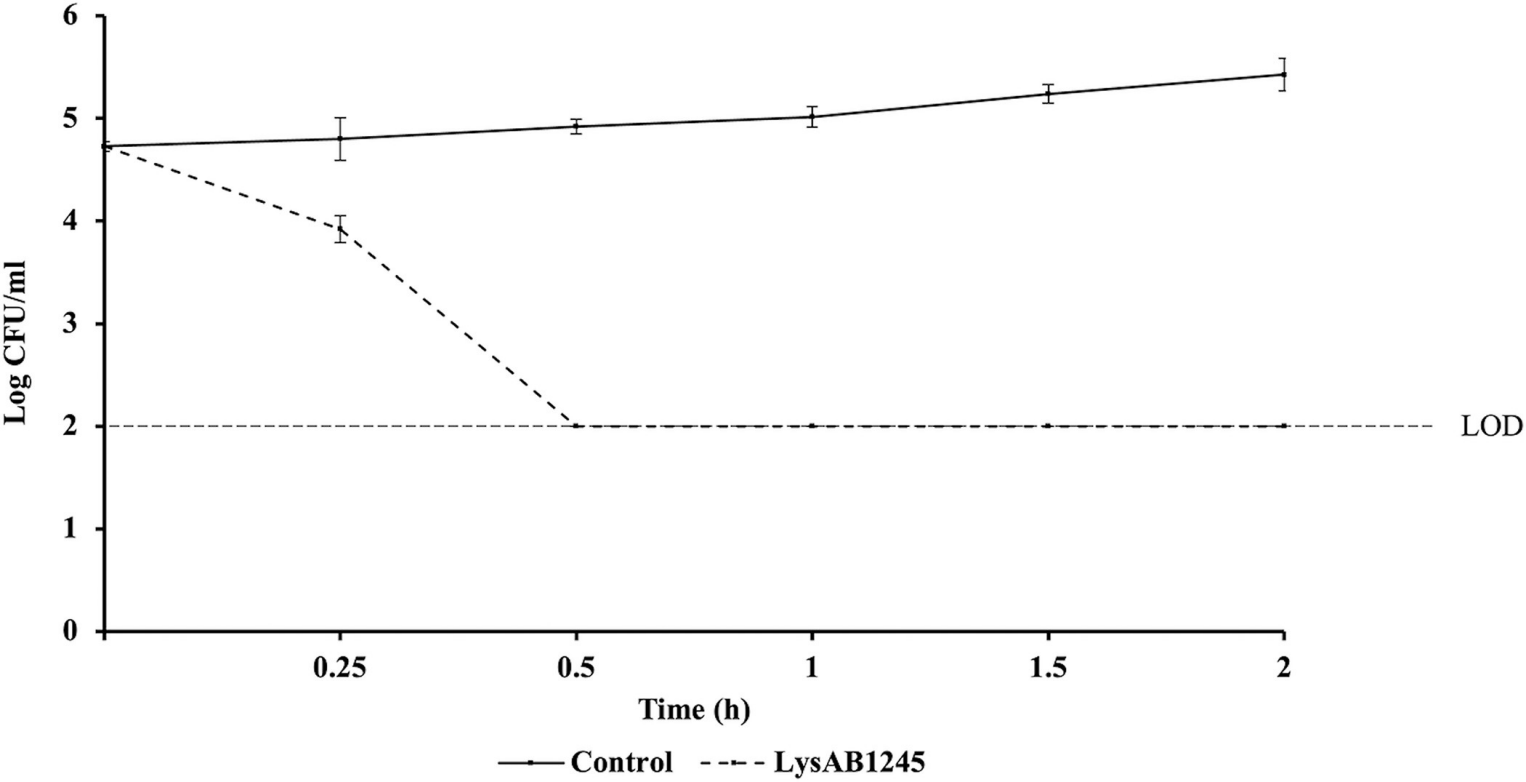
Time-kill curve of *A*. *baumannii* ABMYH-1245 incubated with LysAB1245. Two independent experiments were performed in triplicate and error bars represent the standard deviation. Limit of detection (LOD) for surviving bacterial cells was 2 log CFU/mL.

Killing curve analysis revealed that LysAB1245 at 1.7 unit/reaction displayed rapid bactericidal activity against *A*. *baumannii* ABMYH-1245 (Fig 6). A 2.92 log reduction (>99% reduction) in viable bacteria was observed within 30 min, compared with control (S2 Fig). In this study, the phage T1245-produced endolysin LysAB1245 was successfully expressed and purified using an automated high-throughput protein synthesis system for producing highly pure, stable, and soluble active proteins.

### Antimicrobial activity of LysAB1245

Furthermore, we examined the antimicrobial activity of LysAB1245 against 20 MDR *A*. *baumannii* isolates and ATCC 19606. The MIC value of LysAB1245 was 4.21 μg/mL (0.1 unit/reaction) for all MDR *A*. *baumannii* isolates and *A*. *baumannii* ATCC 19606. The lowest concentration of LysAB1245 with bactericidal activity was 4.21 μg/mL, which was identical to the MIC value (Table 2). In general, the peptidoglycan structure of Gram-negative bacteria is highly conserved. Therefore, the conservation of the peptidoglycan structure among the tested isolates, which serves as the target site of endolysin, might have resulted in the same MIC values of LysAB1245.

**Table 2:** Minimal inhibitory concentrations (MICs) and minimal bactericidal concentrations (MBCs) of LysAB1245 against *Acinetobacter baumannii*.

*Acinetobacter baumannii*	Antibacterial activities (μg/mL)	
	MIC	MBC
20 clinical isolates	4.21	4.21
ATCC 19606	4.21	4.21

According to previous report, no cytotoxic effect of endolysin LysSS was observed on human lung cell line A549 at concentrations less than 250 μg/mL [21]. Additionally, the therapeutic effects of phage endolysins in mouse models of infection have been extensively reported [38,41,42]. For example, the direct administration of endolysin by inhalation improved survival rate by 80% in a mouse model of pneumococcal pneumonia [43]. Moreover, treatment with endolysin SAL200 by inhalation did not induce abnormal inflammatory response in mice with pneumonia [41]. Furthermore, the toxicity and safety of phage endolysin SAL200 administered via intravenous injection has been assessed in tested animals for the drug development process [44]. In 2017, endolysin SL200 was successful administered to target drug-resistant staphylococcal infections in humans [45].

The findings of this study suggest that LysAB1245 would be valuable to further development as a new potential therapeutic alternative for controlling of nosocomial MDR *A*. *baumannii* infections. However, studies are necessary to elucidate the optimal dosage and bactericidal mechanism of LysAB1245 using mammalian infection models before reaching the phase of clinical trials in humans.

## Conclusions

In the present study, the endolysin LysAB1245 from *Acinetobacter* phage T1245 was successfully expressed and purified using an automated protein synthesis system with high-purity target proteins. A novel purified LysAB1245 exhibited a broad lytic spectrum activity against MDR *A*. *baumannii* isolates, which belong to various major capsular types. Additionally, LysAB1245 displayed rapid bactericidal activity and stability under various pH and temperature conditions. This work elucidated a potential of LysAB1245 as a new potential therapeutic agent towards the management of MDR *A*. *baumannii* infections.

## Supporting information

S1 Fig Raw image. SDS-PAGE and western blot images for Fig 2. Lane 1, molecular size marker; lane 2, purified LysAB1245; lane 3, expression sample; lane 4, washed sample; lane 5, unbound sample.
(PDF)

S2 Fig Data was used to build graphs in this reported study. The values used to build the graphs included the means and standard deviations.
(PDF)

S1 File (ZIP)

## References

[1] Perez S, Innes GK, Walters MS, Mehr J, Arias J, Greeley R, et al. Increase in hospital-acquired carbapenem-resistant Acinetobacter baumannii infection and colonization in an acute care hospital during a surge in COVID-19 admissions—New Jersey, February–July 2020. MMWR Morb Mortal Wkly Rep. 2020;69: 1827–1831. doi: 10.15585/mmwr.mm6948e1 33270611 PMC7714028

[2] Boral J, Genç Z, Pınarlık F, Ekinci G, Kuskucu MA, İrkören P, et al. The association between Acinetobacter baumannii infections and the COVID-19 pandemic in an intensive care unit. Sci Rep. 2022;12: 20808. doi: 10.1038/s41598-022-25493-8 36460749 PMC9716169

[3] Russo A, Gavaruzzi F, Ceccarelli G, Borrazzo C, Oliva A, Alessandri F, et al. Multidrug-resistant Acinetobacter baumannii infections in COVID-19 patients hospitalized in intensive care unit. Infection. 2022;50: 83–92. doi: 10.1007/s15010-021-01643-4 34176088 PMC8236000

[4] Zhou H, Yao Y, Zhu B, Ren D, Yang Q, Fu Y, et al. Risk factors for acquisition and mortality of multidrug-resistant Acinetobacter baumannii bacteremia: A retrospective study from a Chinese hospital. Medicine. 2019;98: e14937. doi: 10.1097/MD.0000000000014937 30921191 PMC6456023

[5] Mohd Sazlly Lim S, Zainal Abidin A, Liew SM, Roberts JA, Sime FB. The global prevalence of multidrug-resistance among Acinetobacter baumannii causing hospital-acquired and ventilator-associated pneumonia and its associated mortality: A systematic review and meta-analysis. J Infect. 2019;79: 593–600. doi: 10.1016/j.jinf.2019.09.012 31580871

[6] Motbainor H, Bereded F, Mulu W. Multi-drug resistance of blood stream, urinary tract and surgical site nosocomial infections of Acinetobacter baumannii and Pseudomonas aeruginosa among patients hospitalized at Felegehiwot referral hospital, Northwest Ethiopia: A cross-sectional study. BMC Infect Dis. 2020;20: 92. doi: 10.1186/s12879-020-4811-8 32000693 PMC6993407

[7] Martinez J, Razo-Gutierrez C, Le C, Courville R, Pimentel C, Liu C, et al. Cerebrospinal fluid (CSF) augments metabolism and virulence expression factors in *Acinetobacter baumannii*. Sci Rep. 2021;11: 4737. doi: 10.1038/s41598-021-81714-6 33637791 PMC7910304

[8] Talyansky Y, Nielsen TB, Yan J, Carlino-Macdonald U, Di Venanzio G, Chakravorty S, et al. Capsule carbohydrate structure determines virulence in *Acinetobacter baumannii*. PLOS Pathog. 2021;17: e1009291. doi: 10.1371/journal.ppat.1009291 33529209 PMC7880449

[9] Akoolo L, Pires S, Kim J, Parker D. The Capsule of Acinetobacter baumannii protects against the innate immune response. J Innate Immun. 2022;14: 543–554. doi: 10.1159/000522232 35320810 PMC9485954

[10] Singh JK, Adams FG, Brown MH. Diversity and function of capsular polysaccharide in Acinetobacter baumannii. Front Microbiol. 2018;9: 3301. doi: 10.3389/fmicb.2018.03301 30687280 PMC6333632

[11] Loraine J, Heinz E, Soontarach R, Blackwell GA, Stabler RA, Voravuthikunchai SP, et al. Genomic and phenotypic analyses of Acinetobacter baumannii isolates from three tertiary care hospitals in Thailand. Front Microbiol. 2020;11: 548. doi: 10.3389/fmicb.2020.00548 32328045 PMC7153491

[12] Hsieh YC, Wang SH, Chen YY, Lin TL, Shie SS, Huang CT, et al. Association of capsular types with carbapenem resistance, disease severity, and mortality in *Acinetobacter baumannii*. Emerg Microbes Infect. 2020;9: 2094–2104. doi: 10.1080/22221751.2020.1822757 32912064 PMC7534287

[13] Kishk R, Soliman N, Nemr N, Eldesouki R, Mahrous N, Gobouri A, et al. Prevalence of aminoglycoside resistance and aminoglycoside modifying enzymes in Acinetobacter baumannii among intensive care unit patients, Ismailia, Egypt. Infect Drug Resist. 2021;14: 143–150. doi: 10.2147/IDR.S290584 33519215 PMC7838519

[14] Liu C, Chen K, Wu Y, Huang L, Fang Y, Lu J, et al. Epidemiological and genetic characteristics of clinical carbapenem-resistant Acinetobacter baumannii strains collected countrywide from hospital intensive care units (ICUs) in China. Emerg Microbes Infect. 2022;11: 1730–1741. doi: 10.1080/22221751.2022.2093134 35730377 PMC9258068

[15] Seleim SM, Mostafa MS, Ouda NH, Shash RY. The role of pmrCAB genes in colistin-resistant Acinetobacter baumannii. Sci Rep. 2022;12: 20951. doi: 10.1038/s41598-022-25226-x 36470921 PMC9722906

[16] Kabic J, Novovic K, Kekic D, Trudic A, Opavski N, Dimkic I, et al. Comparative genomics and molecular epidemiology of colistin-resistant *Acinetobacter baumannii*. Comput Struct Biotechnol J. 2023;21: 574–585. doi: 10.1016/j.csbj.2022.12.045 36659926 PMC9816908

[17] Pormohammad A, Mehdinejadiani K, Gholizadeh P, Nasiri MJ, Mohtavinejad N, Dadashi M, et al. Global prevalence of colistin resistance in clinical isolates of *Acinetobacter baumannii*: A systematic review and meta-analysis. Microb Pathog. 2020;139: 103887. doi: 10.1016/j.micpath.2019.103887 31765766

[18] Sun B, Liu H, Jiang Y, Shao L, Yang S, Chen D. New mutations involved in colistin resistance in Acinetobacter baumannii. mSphere. 2020;5: e00895–19. doi: 10.1128/mSphere.00895-19 32238571 PMC7113586

[19] Tan X, Chen H, Zhang M, Zhao Y, Jiang Y, Liu X, et al. Clinical experience of personalized phage therapy against carbapenem-resistant *Acinetobacter baumannii* lung infection in a patient with chronic obstructive pulmonary disease. Front Cell Infect Microbiol. 2021;11: 631585. doi: 10.3389/fcimb.2021.631585 33718279 PMC7952606

[20] Wu N, Dai J, Guo M, Li J, Zhou X, Li F, et al. Pre-optimized phage therapy on secondary *Acinetobacter baumannii* infection in four critical COVID-19 patients. Emerg Microbes Infect. 2021;10: 612–618. doi: 10.1080/22221751.2021.1902754 33703996 PMC8032346

[21] Kim S, Lee DW, Jin JS, Kim J. Antimicrobial activity of LysSS, a novel phage endolysin, against Acinetobacter baumannii and Pseudomonas aeruginosa. J Glob Antimicrob Resist. 2020;22: 32–39. doi: 10.1016/j.jgar.2020.01.005 32006750

[22] Gouveia A, Pinto D, Veiga H, Antunes W, Pinho MG, são-José C. Synthetic antimicrobial peptides as enhancers of the bacteriolytic action of staphylococcal phage endolysins. Sci Rep. 2022;12: 1245. doi: 10.1038/s41598-022-05361-1 35075218 PMC8786859

[23] Briers Y, Schmelcher M, Loessner MJ, Hendrix J, Engelborghs Y, Volckaert G, et al. The high-affinity peptidoglycan binding domain of *Pseudomonas* phage endolysin KZ144. Biochem Biophys Res Commun. 2009;383: 187–191. doi: 10.1016/j.bbrc.2009.03.161 19348786

[24] Wu M, Hu K, Xie Y, Liu Y, Mu D, Guo H, et al. A novel phage PD-6A3, and its endolysin Ply6A3, with extended lytic activity against Acinetobacter baumannii. Front Microbiol. 2018;9: 3302. doi: 10.3389/fmicb.2018.03302 30687281 PMC6333635

[25] Rahman MU, Wang W, Sun Q, Shah JA, Li C, Sun Y, et al. Endolysin, a promising solution against antimicrobial resistance. Antibiotics (Basel). 2021;10: 1277. doi: 10.3390/antibiotics10111277 34827215 PMC8614784

[26] Gerova M, Halgasova N, Ugorcakova J, Bukovska G. Endolysin of bacteriophage BFK20: Evidence of a catalytic and a cell wall binding domain. FEMS Microbiol Lett. 2011;321: 83–91. doi: 10.1111/j.1574-6968.2011.02312.x 21592196

[27] Chang Y, Ryu S. Characterization of a novel cell wall binding domain-containing *Staphylococcus aureus* endolysin LysSA97. Appl Microbiol Biotechnol. 2017;101: 147–158. doi: 10.1007/s00253-016-7747-6 27498125

[28] Soontarach R, Srimanote P, Enright MC, Blundell-Hunter G, Dorman MJ, Thomson NR, et al. Isolation and characterisation of bacteriophage selective for key Acinetobacter baumannii capsule chemotypes. Pharmaceuticals (Basel). 2022;15: 443. doi: 10.3390/ph15040443 35455440 PMC9027227

[29] Sambrook J, Fritsch EF, Maniatis. Molecular Cloning: A Laboratory Manual, 2nd (ed.). New York: Cold Spring Harbor Laboratory Press; 1989.

[30] Lai MJ, Lin NT, Hu A, Soo PC, Chen LK, Chen LH, et al. Antibacterial activity of Acinetobacter baumannii phage ϕAB2 endolysin (LysAB2) against both Gram-positive and gram-negative bacteria. Appl Microbiol Biotechnol. 2011;90: 529–539. doi: 10.1007/s00253-011-3104-y 21264466

[31] Clinical and Laboratory Standards Institute (CLSI). Performance standards for antimicrobial susceptibility testing. 30th ed. CLSI Document m100. Wayne (Pennsylvania): CLSI; 2020.

[32] Simpson DJ, Sacher JC, Szymanski CM. Exploring the interactions between bacteriophage-encoded glycan binding proteins and carbohydrates. Curr Opin Struct Biol. 2015;34: 69–77. doi: 10.1016/j.sbi.2015.07.006 26275959

[33] Larpin Y, Oechslin F, Moreillon P, Resch G, Entenza JM, Mancini S. In vitro characterization of PlyE146, a novel phage lysin that targets Gram-negative bacteria. PLOS ONE. 2018;13: e0192507. doi: 10.1371/journal.pone.0192507 29408864 PMC5800649

[34] Huang G, Shen X, Gong Y, Dong Z, Zhao X, Shen W, et al. Antibacterial properties of Acinetobacter baumannii phage Abp1 endolysin (PlyAB1). BMC Infect Dis. 2014;14: 681. doi: 10.1186/s12879-014-0681-2 25495514 PMC4274762

[35] Thummeepak R, Kitti T, Kunthalert D, Sitthisak S. Enhanced antibacterial activity of Acinetobacter baumannii bacteriophage ØABP-01 endolysin (LysABP-01) in combination with colistin. Front Microbiol. 2016;7: 1402. doi: 10.3389/fmicb.2016.01402 27656173 PMC5013039

[36] Jiang Y, Xu D, Wang L, Qu M, Li F, Tan Z, et al. Characterization of a broad-spectrum endolysin LysSP1 encoded by a *Salmonella* bacteriophage. Appl Microbiol Biotechnol. 2021;105: 5461–5470. doi: 10.1007/s00253-021-11366-z 34241646

[37] Ni P, Wang L, Deng B, Jiu S, Ma C, Zhang C, et al. Characterization of a lytic bacteriophage against Pseudomonas syringae pv. actinidiae and its endolysin. Viruses. 2021;13: 631. doi: 10.3390/v13040631 33917076 PMC8067700

[38] Lood R, Winer BY, Pelzek AJ, Diez-Martinez R, Thandar M, Euler CW, et al. Novel phage lysin capable of killing the multidrug-resistant gram-negative bacterium *Acinetobacter baumannii* in a mouse bacteremia model. Antimicrob Agents Chemother. 2015;59: 1983–1991. doi: 10.1128/AAC.04641-14 25605353 PMC4356752

[39] Düring K, Porsch P, Mahn A, Brinkmann O, Gieffers W. The non‐enzymatic microbicidal activity of lysozymes. FEBS Lett. 1999;449: 93–100. doi: 10.1016/s0014-5793(99)00405-6 10338111

[40] Chen J, Li G, Wan F, Liu P, Du F, Shao Y, et al. Virulence characteristics and drug resistance of the prevalent capsule types in *Acinetobacter baumannii*. Microb Drug Resist. 2023;29: 274–279. doi: 10.1089/mdr.2022.0310 37074067

[41] Bae JY, Jun KI, Kang CK, Song KH, Choe PG, Bang JH, et al. Efficacy of intranasal administration of the recombinant endolysin SAL200 in a lethal murine *Staphylococcus aureus* pneumonia model. Antimicrob Agents Chemother. 2019;63: e02009–18. doi: 10.1128/AAC.02009-18 30670417 PMC6437543

[42] Raz A, Serrano A, Hernandez A, Euler CW, Fischetti VA. Isolation of phage lysins that effectively kill *Pseudomonas aeruginosa* in mouse models of lung and skin infection. Antimicrob Agents Chemother. 2019;63: e00024–19. doi: 10.1128/AAC.00024-19 31010858 PMC6591642

[43] Doehn JM, Fischer K, Reppe K, Gutbier B, Tschernig T, Hocke AC, et al. Delivery of the endolysin cpl-1 by inhalation rescues mice with fatal pneumococcal pneumonia. J Antimicrob Chemother. 2013;68: 2111–2117. doi: 10.1093/jac/dkt131 23633685

[44] Jun SY, Jung GM, Yoon SJ, Choi YJ, Koh WS, Moon KS, et al. Preclinical safety evaluation of intravenously administered SAL200 containing the recombinant phage endolysin SAL-1 as a pharmaceutical ingredient. Antimicrob Agents Chemother. 2014;58: 2084–2088. doi: 10.1128/AAC.02232-13 24449776 PMC4023757

[45] Jun SY, Jang IJ, Yoon S, Jang K, Yu KS, Cho JY, et al. Pharmacokinetics and tolerance of the phage endolysin-based candidate drug SAL200 after a single intravenous administration among healthy volunteers. Antimicrob Agents Chemother. 2017;61: e02629–16. doi: 10.1128/AAC.02629-16 28348152 PMC5444177

