# Supplementary material for: Characterization of a novel bacteriophage endolysin (LysAB1245) with extended lytic activity against distinct capsular types associated with Acinetobacter baumannii resistance
Source: PLoS One. 2024 Jan 2;19(1):e0296453. doi: 10.1371/journal.pone.0296453 (PMC10760713; doi:10.1371/journal.pone.0296453)
Supplement: S1 Fig — SDS-PAGE and western blot images for Fig 2. Lane 1, molecular size marker; lane 2, purified LysAB1245; lane 3, expression sample; lane 4, washed sample; lane 5, unbound sample. (PDF) [file pone.0296453.s001.pdf]

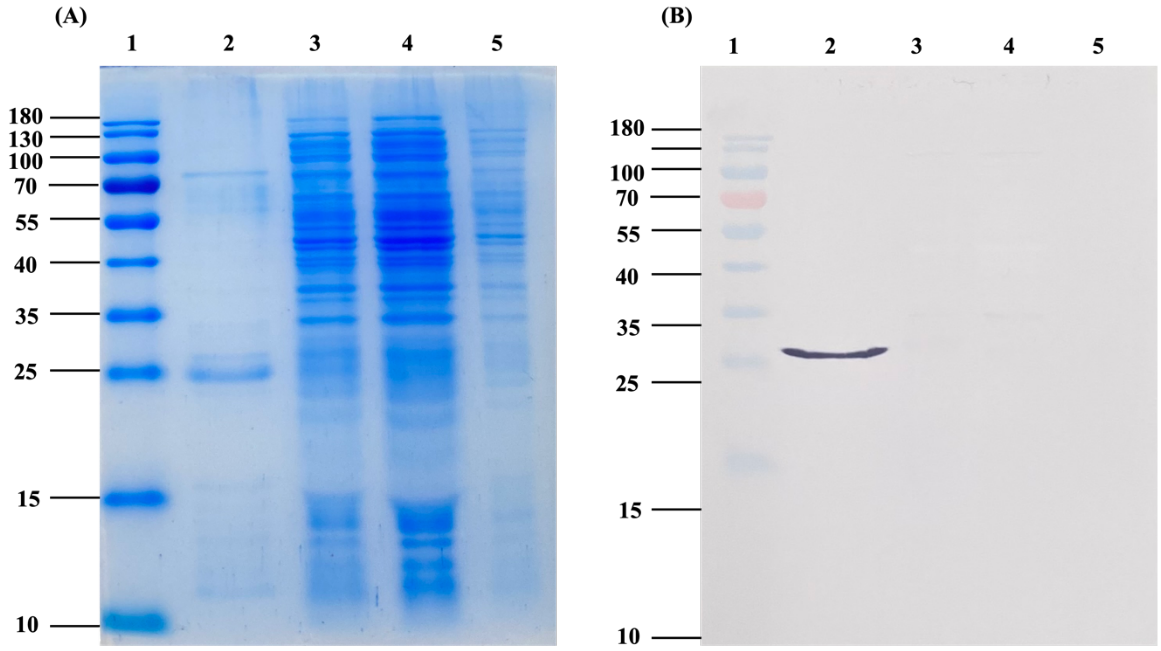

S1 Fig. Raw image. SDS-PAGE and western blot images for Fig 2. Lane 1, molecular size marker; lane 2, purified LysAB1245; lane 3, expression sample; lane 4, washed sample; lane 5, unbound sample.
