# Supplementary material for: Characterization of a novel bacteriophage endolysin (LysAB1245) with extended lytic activity against distinct capsular types associated with Acinetobacter baumannii resistance
Source: PLoS One. 2024 Jan 2;19(1):e0296453. doi: 10.1371/journal.pone.0296453 (PMC10760713; doi:10.1371/journal.pone.0296453)
Supplement: S2 Fig — The values used to build the graphs included the means and standard deviations. (PDF) [file pone.0296453.s002.pdf]

lolysin activity on ABMYS-1	CFU/ml	CFU/ml	CFU/ml	log	log	log	SD
LysAB1245	1	2	3	1	2	3	
0m	1.70E+05	1.50E+05	1.40E+05	5.23	5.18	5.15	0.04
2h	1.00E+02	1.00E+02	1.00E+02	2.00	2.00	2.00	0.00
24h	1.00E+02	1.00E+02	1.00E+02	2.00	2.00	2.00	0.00
**PBS**							
0m	1.50E+05	1.90E+05	1.50E+05	5.18	5.28	5.18	0.05
2h	2.10E+05	2.40E+05	2.80E+05	5.32	5.38	5.45	0.06
24h	1.20E+06	1.30E+06	1.80E+06	6.08	6.11	6.26	0.08
**pH stability**	**acterial survival (%**	**cterial survival (%**	**acterial survival (%**	**acterial reductio**	**acterial reductio**	**acterial reducti**	**SD**
	1	2	3	1	2	3	
Control(PBS7.4)	100.00	100.00	100.00				
Lys4.5	0.01	0.00	0.01	99.99	100.00	99.99	0.00
Lys5.5	0.01	0.00	0.01	99.99	100.00	99.99	0.00
Lys7.4	0.01	0.00	0.01	99.99	100.00	99.99	0.00
Lys8.5	0.01	0.00	0.01	99.99	100.00	99.99	0.00
Lys10.5	0.01	0.00	0.01	99.99	100.00	99.99	0.00
**Thermal stability**	**acterial survival (%**	**cterial survival (%**	**acterial survival (%**	**acterial reductio**	**acterial reductio**	**acterial reducti**	**SD**
	1	2	3	1	2	3	
Control(PBS)	100.00	100	100				
4	0.88	0.83	0.89	99.13	99.17	99.11	0.03
25	1.13	1.00	1.00	98.88	99.00	99.00	0.06
37	1.06	1.11	1.00	98.94	98.89	99.00	0.05
50	1.63	1.50	1.58	98.38	98.50	98.42	0.05
60	1.75	1.61	1.58	98.25	98.39	98.42	0.08
70	1.69	1.72	1.74	98.31	98.28	98.26	0.02
**Time-kill**	**CFU/ml**	**CFU/ml**	**CFU/ml**	**log**	**log**	**log**	**SD**
**ontrol (PBS) at interval tim**	1	2	3	1	2	3	
0	5.00E+04	5.00E+04	6.00E+04	4.70	4.70	4.78	0.05
0.25	4.00E+04	5.00E+04	1.00E+05	4.60	4.70	5.00	0.21
0.5	3.00E+04	4.00E+04	4.00E+04	4.48	4.60	4.60	0.07
1	4.00E+04	4.00E+04	6.00E+04	4.60	4.60	4.78	0.10
1.5	8.00E+04	9.00E+04	1.20E+05	4.90	4.95	5.08	0.09
2	8.00E+04	8.00E+04	1.50E+05	4.90	4.90	5.18	0.16
2.5	1.60E+05	1.80E+05	1.80E+05	5.20	5.26	5.26	0.03
	**CFU/ml**	**CFU/ml**	**CFU/ml**	**log**	**log**	**log**	**SD**
**LysAB1245 at interval time**	1	2	3	1	2	3	
0	5.00E+04	6.00E+04	5.00E+04	4.70	4.78	4.70	0.05
0.25	6.00E+03	8.00E+03	1.10E+04	3.78	3.90	4.04	0.13
0.5	1.00E+02	1.00E+02	1.00E+02	2.00	2.00	2.00	0.00
1	1.00E+02	1.00E+02	1.00E+02	2.00	2.00	2.00	0.00
1.5	1.00E+02	1.00E+02	1.00E+02	2.00	2.00	2.00	0.00
2	1.00E+02	1.00E+02	1.00E+02	2.00	2.00	2.00	0.00
2.5	1.00E+02	1.00E+02	1.00E+02	2.00	2.00	2.00	0.00
